# Animal Source Food Social and Behavior Change Communication Intervention Among Girinka Livestock Transfer Beneficiaries in Rwanda: A Cluster Randomized Evaluation

**DOI:** 10.9745/GHSP-D-21-00082

**Published:** 2021-09-30

**Authors:** Valerie L. Flax, Emily Ouma, Lambert Izerimana, Mary-Ann Schreiner, Alice O. Brower, Eugene Niyonzima, Carine Nyilimana, Adeline Ufitinema, Agnes Uwineza

**Affiliations:** a RTI International, Research Triangle Park, NC, USA.; b International Livestock Research Institute, Kampala, Uganda.; c Three Stones International, Kigali, Rwanda.; d University of Rwanda, Department of Food Science and Technology, Musanze, Rwanda.; e Ministry of Agriculture and Animal Resources, Kigali, Rwanda.; f Ministry of Gender and Family Promotion, National Childhood Development Agency, Kigali, Rwanda.

## Abstract

Animal source foods (ASFs), including cow’s milk, contain essential nutrients and contribute to a healthy diet, but frequency of intake is low among children in low- and middle-income countries. We hypothesized that an ASF social and behavior change communication (SBCC) intervention implemented by community health workers (CHWs) would increase child milk consumption and dietary diversity in households that received a cow from the Government of Rwanda’s Girinka livestock transfer program. We tested the 9-month SBCC intervention among children aged 12–29 months at baseline in administrative cells randomly assigned to the intervention or control. Most mothers in the intervention group were exposed to CHWs’ home visits (90.7%) or community-level activities (82.8%). At endline, more mothers in the intervention group compared with the control group knew that cow’s milk was an ASF (90.1% vs. 81.7%, *P*=.03) and could be introduced to children at 12 months (41.7% vs. 18.7%, *P*<.001). More mothers in the intervention group compared with the control group knew they should feed their children ASFs (76.2% vs. 62.1%, *P*=.01) and give them 1 cup of cow’s milk per day (20.6% vs. 7.8%, *P*<.001). Children’s consumption of fresh cow’s milk 2 or more times per week increased in the intervention group, although not significantly (8.0 percentage points, *P*=.17); minimum dietary diversity was unchanged. Children in the intervention group had increased odds of consuming cow’s milk 2 or more times per week if their mothers recalled hearing that children should drink 1 cup of cow’s milk per day during a CHW’s home visit [odds ratio (OR) 2.1, 95% confidence interval (CI) (1.1, 3.9)] or a community activity [OR 2.0, 95% CI (1.2, 3.5)]. Approximately half of the children had no milk during the past week because their households produced too little or sold what was produced. In poor households receiving a livestock transfer, strategies to further tailor SBCC and increase cow’s milk production may be needed to achieve larger increases in children’s frequency of milk consumption.

## Abstract (precis)

A social and behavior change communication intervention designed to promote consumption of cow’s milk among families that received a cow from a government livestock transfer program increased mothers’ knowledge and awareness of milk consumption. Although intervention exposure was associated with increased frequency of children’s cow’s milk intake, it did not lead to increased consumption or dietary diversity.

## Teaser

**Key Findings:**

- A social and behavior change communication (SBCC) intervention was associated with increased odds of children consuming cow’s milk 2 or more times per week.
- For approximately half of the children, frequency of cow’s milk consumption was limited by inadequate household milk production or sale of the milk produced.
- SBCC did not influence the percentage of households that kept or sold their milk, demonstrating that SBCC alone is not enough to change nutrition outcomes in households with poor food security.

**Key Implications:**

- Community health workers successfully implemented the intervention and the SBCC messages have been incorporated into the recently revised national CHW counseling cards.
- SBCC for this target population should be implemented for a longer period and tailored to discuss financial management and dietary choices with a limited budget. Accompanying training or other activities to assist households that receive cows are needed to ensure adequate cow’s milk production for home consumption.
- High levels of severe food insecurity in this population may have limited the potential of the SBCC to improve dietary diversity and more substantially improve frequency of cow’s milk consumption.

Résumé en français à la fin de l'article.

## INTRODUCTION

Animal source foods (ASFs), including milk, are a rich source of energy, protein, and micronutrients and can contribute to a healthy and diverse diet in young children.^1^ Yet consumption of ASFs by young children in low- and middle-income countries (LMICs) is limited. Based on recent nationally representative data from countries in East and Southern Africa, only 49% of children aged 6–23 months consumed at least 1 ASF on the day before the survey and 19% consumed dairy, which are the lowest prevalence estimates across LMIC regions.^2^

Consumption of ASFs by young children in LMICs is influenced by several different factors, including affordability, accessibility, child’s age, perceived nutritional value, taste preferences, and sociocultural factors (e.g., food prohibitions, preferential food allocation, child feeding styles).^3^^–^^5^ Livestock ownership is also related to ASF consumption by children, in part, because it increases ASF accessibility and income.^6^^–^^9^ In sub-Saharan Africa, children in livestock-owning or pastoral households are more likely to consume ASFs than those in non-livestock-owning households,^5^^,^^10^^–^^12^ and children in families that received a livestock transfer or participated in a livestock production program also consume more ASFs than those that have not participated in such programs.^13^^–^^16^ However, in nonpastoral, livestock-owning households in sub-Saharan Africa, consumption of ASFs is suboptimal because livestock are kept for selling or are considered monetary assets or because consumption of staple foods uses fewer resources, so it is prioritized over ASF consumption.^17^^–^^21^

One of the pathways through which agriculture programs, such as livestock transfers, can have an impact on the consumption of nutritious foods, such as ASFs, and child nutritional status is the “own production to consumption” pathway.^22^^,^^23^ This pathway is based on the theory that household food production leads to consumption of ASFs, leading to better nutrient intake and positive nutritional outcomes, including for children. Food production, expenditures, and consumption can be influenced and increased by social and behavior change communication (SBCC),^23^^–^^25^ including group sessions, home visits, community meetings, and mass media. Child consumption of ASFs and subsequently their nutritional status are increased in households where SBCC is incorporated into livestock production interventions.^23^^,^^26^

Child consumption of ASFs and subsequently their nutritional status are increased in households where SBCC is incorporated into livestock production interventions.

The Government of Rwanda’s One Cow per Poor Family Girinka program is a presidential initiative started in 2006 to provide an exotic or cross-bred dairy cow to households with low socioeconomic status that do not already own cattle.^27^ Economic eligibility for the program is based on the government’s *Ubudehe* or socioeconomic classification categories, which are updated every 3 years.^28^ The goals of the Girinka program are to increase social cohesion and integration and to improve income, food security, and nutrition in poor households. Previous evaluations showed the economic benefits of the Girinka program,^29^^,^^30^ but the nutrition benefits are less clear, despite the program being implemented in a context with high stunting prevalence (38%) and low milk consumption (21%) among young children.^31^ The Girinka program does not include a nutrition education or SBCC component promoting the consumption of home-produced milk.

To address this gap, we conducted a cluster-randomized trial to test an SBCC intervention to increase cow’s milk consumption among Girinka households with a young child. The study aimed to evaluate whether training community health workers (CHWs) to conduct community and household SBCC activities promoting cow’s milk consumption would increase milk consumption and dietary diversity among young children in households that had received a cow through the Girinka program.

## METHODS

### Study Overview

This cluster-randomized controlled trial was designed to test the impact of an SBCC intervention to promote the consumption of ASFs, especially cow’s milk, on maternal ASF knowledge and awareness and on child milk consumption and dietary diversity in households that had received a cow from the Girinka program. The trial was registered at ClinicalTrials.gov (NCT03455647).

This study tested the impact of an SBCC intervention promoting consumption of ASFs on maternal ASF knowledge and on child milk consumption and dietary diversity.

The study was conducted in Nyabihu and Ruhango Districts, Rwanda. The districts were selected in consultation with the Ministry of Local Government to include districts with a high prevalence of childhood stunting and poverty.^31^^,^^32^ Districts in Rwanda are subdivided administratively into sectors, which are further divided into cells. Cells typically contain 5–7 villages, but they can range from 4 to 12 villages.

### Sample Selection and Sample Size

We randomly assigned administrative cells in the 2 districts to intervention or control. Nyabihu had nutrition programs in different parts of the district, whereas Ruhango had nutrition programs operating throughout the district. Therefore, randomization in Nyabihu was stratified by ongoing nutrition programs. The existing nutrition programs in the counties did not specifically promote ASF or cow’s milk consumption by young children. In both districts, the randomized cells were balanced on total population size.

We obtained lists of households that had received a cow through the Girinka program from district and sector animal resources officers. Households were eligible for enrollment at baseline if they received a Girinka cow in 2017 or earlier or a Girinka calf in 2016 or earlier, the animal was still alive, the mother was 18–49 years of age and had a child who was 12–29 months of age, and the biological mother lived with the child.

Our target was 4 households per cell. However, because we had challenges finding enough eligible households and many cells had fewer than 4 eligible households, we included up to 9 households per cell. If a cell contained more than 9 eligible households, the data collection team randomly selected from among those that were eligible.

We calculated sample sizes for 2 child outcomes—minimum dietary diversity (consumption of ≥4 food groups in the past 24 hours) and milk consumption in the past 24 hours—based on a comparison of the changes in these parameters between baseline and endline. Minimum dietary diversity required a larger sample size (Supplement Table 1), so it was used as the sample size for the study. To detect a 15-percentage point difference between groups in the prevalence of minimum dietary diversity^33^ (i.e., at endline: control 29% and intervention 44%) with 80% power and alpha=0.05, required 208 households per group, assuming an average cluster size of 4 households per cell, an intracluster correlation of 0.10, and a design effect of 1.3. We added 10% to the sample to account for attrition, resulting in 229 households per group and a total baseline sample size of 458.

### Intervention

**FIGURE 1:**
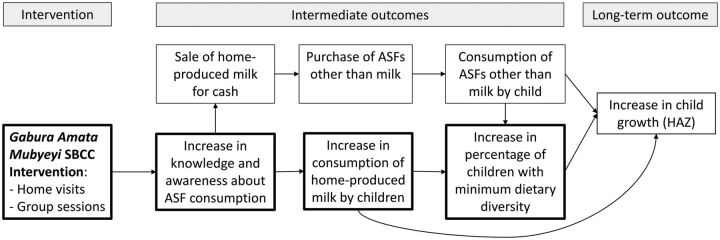
Theory of Change for the *Gabura Amata Mubyeyi* Social and Behavior Change Communication Intervention to Promote Consumption of Cow’s Milk Among Children, Rwanda Abbreviations: ASF, animal source food; HAZ, height-for-age z-score; SBCC, social and behavior change communication.

The SBCC intervention was known as *Gabura Amata Mubyeyi* in Kinyarwanda, which translates to “Parents, Give Milk” in English. The intervention was developed based on formative research and guided by the theory of change shown in Figure 1. The theory of change posits that appropriate and effective SBCC on ASF consumption from CHWs reaches mothers and increases their knowledge. Mothers are concerned about child nutrition and are willing and able to adopt the recommended practices. They increase the child’s consumption of home-produced milk from their Girinka cow, which in turn increases child dietary diversity and may contribute over the long term to increases in child growth directly or through improved dietary diversity. In this analysis, we measured the effects of the intervention on the intermediate outcomes in the own-production pathway indicated in bold boxes in Figure 1. The theory of change also shows an alternate pathway to increased dietary diversity and growth through the purchase of ASFs.

**Figure:**
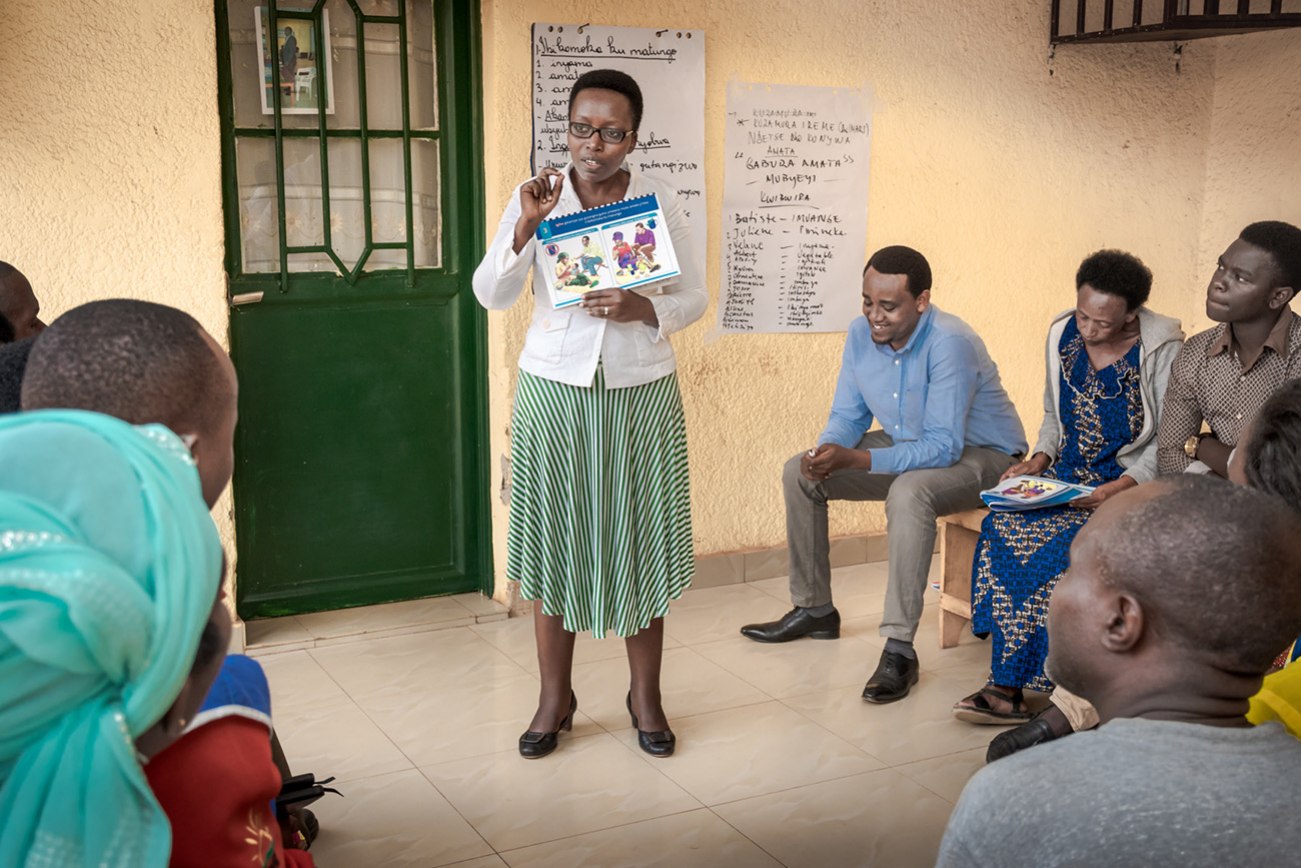
Three Stones International coordinator training community health workers. © 2019 Jean Claude Gasangwa/Three Stones International

The SBCC intervention was known as *Gabura Amata Mubyeyi* in Kinyarwanda, which translates to “Parents, Give Milk” in English.

The intervention and SBCC materials were designed in collaboration with the National Child Development Agency, which coordinates nutrition activities in Rwanda. The SBCC materials consisted of counseling cards, a poster, and a brochure translated into Kinyarwanda. The counseling cards were designed using the same style as the Rwanda maternal, infant, and young child nutrition counseling cards. The messages from the SBCC materials related to this analysis are shown in the Box. Rwanda does not have food-based dietary guidelines, so the recommendation in this study to give children 1 cup of milk per day was based on the Rwanda Agriculture Board’s One Cup of Milk per Child program.^34^ This quantity of milk is low compared with the U.S. Department of Agriculture dairy recommendations for children 12–23 months (1 2/3 to 2 cups) and 2–3 years (2 to 2 1/2 cups).^35^ The recommendation to introduce cow’s milk to the child’s diet at 12 months is based on evidence that cow’s milk can result in occult blood loss from infants’ gastrointestinal tracts^36^ and the inability of infants’ kidneys to handle the high levels of protein, sodium, and potassium in cow’s milk.^37^

Community and environmental health officers, who supervise CHWs, were trained to train CHWs to use the SBCC materials and conduct household and community SBCC sessions. The household visits were specifically targeted at households included in the intervention arm of the study. The community sessions were offered to all community members in the intervention cells. The intervention was implemented from February to October 2019 and was designed as an addition to CHWs’ usual activities. CHWs were asked to visit households in the SBCC intervention group monthly and conduct community SBCC sessions monthly. At the time of this study, SBCC materials specifically promoting ASF consumption were not available to CHWs through the government or its implementing partners. In the 2 study districts, only CHWs in the intervention group had copies of the *Gabura Amata Mubyeyi* SBCC materials. CHWs work within their own administrative cells, so the possibility of the intervention being inadvertently implemented outside the target cells was very low.

### Data Collection

Experienced enumerators were trained to conduct the baseline and endline surveys. The training covered screening and enrollment, consent procedures, review of the questionnaire on paper and in Open Data Kit (ODK), and a pilot. Enumerators collected the data at the participants’ households using tablets with the questionnaire programmed in ODK. Completed interviews were reviewed by the field supervisor and uploaded to a secure server. The baseline survey was conducted in batches in April–May, July–August, and October–November 2018 as the lists of Girinka participants were received. The bulk of the endline survey was conducted from January–March 2020; 6 participants had their interviews in July 2020 because of travel restrictions related to COVID-19.

The questionnaires were developed in English then translated into Kinyarwanda. They included questions on child diet and feeding practices from the World Health Organization (WHO) infant and young child feeding questionnaire,^38^ including the types of fluids and foods the child consumed in the past 24 hours (24-hour recall). The questionnaire also collected information on the frequency of the child’s consumption of cow’s milk and other ASFs in the past 7 days (7-day recall), maternal knowledge and awareness related to milk, participation in nutrition activities conducted by CHWs, household food insecurity, livestock ownership, household milk production, and socioeconomic characteristics. Maternal ASF knowledge questions were asked without providing response options, whereas maternal awareness was gauged by asking women if they had ever heard about specific practices. Questions on general exposure to home visits and community activities conducted by CHWs were asked to participants in both study groups at baseline and endline. The endline questionnaire also included questions on intervention exposure for participants in the intervention group only. Intervention exposure questions were posed in a yes/no format, except for questions about the numbers of home visits or community activities attended.

### Data Analysis

Several variables in this study were calculated or derived from the data. Child dietary diversity was calculated using the WHO infant and young child feeding indicator guidelines.^38^ We did not use the updated dietary diversity indicator that includes breast milk because part of our study population was ≥24 months at baseline and most children were ≥24 months at endline and no longer breastfeeding. Household food insecurity access categories were calculated using guidelines from the FANTA project.^39^ The household domestic asset index was calculated for all movable assets including livestock, using guidelines by Njuki et al.^40^ Each of the assets was assigned a weight, which was then adjusted for the age of the asset. Higher asset scores indicate higher socioeconomic status. The household land asset was calculated as total agricultural land parcels owned by the household in square meters. A CASHPOR housing index that captures the quality of housing in terms of roof, wall, and floor materials was used as a proxy for measuring poverty.^40^ CASHPOR scores below 5 indicate very poor housing and scores from 5 to 9 indicate poor housing.

We used longitudinal random effects regression models with robust standard errors in Stata (MP, version 16.0) to account for clustering at the level of the cell and estimate difference-in-difference for the impact of the SBCC intervention on child milk consumption (24-hour recall and 7-day recall) and minimum dietary diversity. We calculated unadjusted difference-in-difference estimates and performed an analysis adjusted for factors that could influence the outcomes (child’s age, child’s sex, current breastfeeding status, mother’s educational status, and mother’s marital status). We calculated the average means or percentages across districts by study group for outcome, socioeconomic, and other variables and used regression models to evaluate the difference in means.

## RESULTS

### Study Participants and Characteristics

**FIGURE 2:**
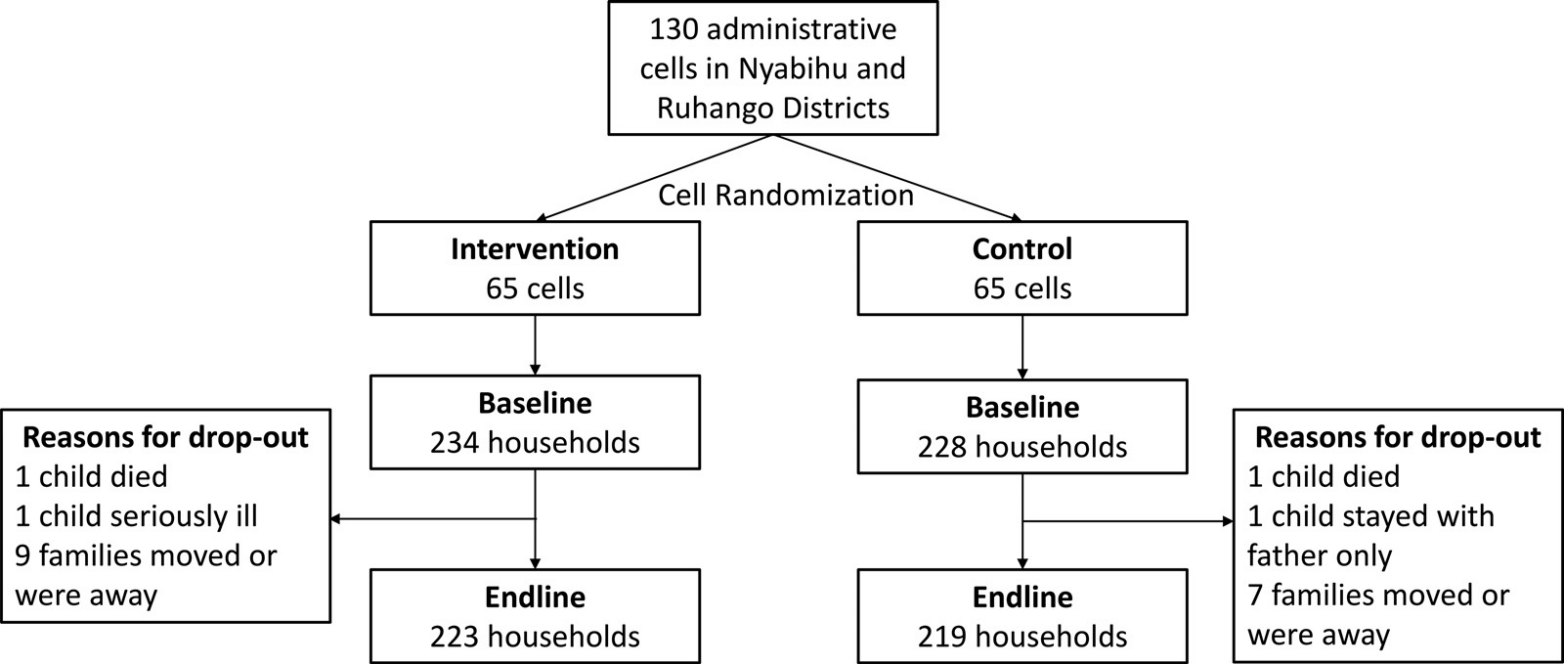
Study Flow Diagram for Participants Involved in a Social and Behavior Change Communication Intervention to Promote Consumption of Cow’s Milk Among Children, Rwanda

The flow of study participants is shown in Figure 2. Less than 5% of participants in both study groups were lost to follow-up between baseline and endline. The main reason for loss to follow-up was families moving away from the area or traveling at the time of endline data collection.

At baseline, fathers in intervention households were older (*P*<.001) and intervention households had a slightly lower mean CASHPOR housing index (*P*=.03) than control households (Table 1). We found no statistically significant differences in other individual and household characteristics of participants in the 2 study groups. In both groups, households contained approximately 6 members on average. Mothers’ mean age was approximately 33 years and children’s mean age was 19 months. About three-quarters of mothers’ had a primary-level education or lower. Households in both groups had very small landholdings and few domestic assets and were living in houses classified as very poor or poor. More than 60% were classified as having severe food insecurity.

**TABLE 1.** Participants’ Individual and Household Characteristics at Baseline

	Intervention (N=234), Mean±SE or %	Control (N=228), Mean±SE or %	*P* Value
No. of household members	5.9±0.1	5.8±0.1	.96
Age of mother, years	33.7±0.4	32.8±0.5	.20
Age of father, years	40.1±1.1	37.6±1.0	.00
No. of children	3.5±0.1	3.4	.78
Age of index child, months	19.6±0.4	19.9±0.4	.97
Sex of index child, % male	46.6	46.9	.71
Marital status of mother			.76
Single	23.2	29.4	
Married	72.1	66.7	
Widowed	2.1	1.8	
Separated/divorced	2.6	2.2	
Index mothers who are household heads, %	5.6	6.6	
Mother’s occupation			.74
Farmer	95.7	92.5	
Housewife	0.4	1.3	
Jobless	1.7	2.2	
Other	2.1	3.9	
Mother’s education			.06
Informal education, never attended school	12.0	14.9	
Lower primary (1–4)	36.8	36.4	
Upper primary (5–8)	39.3	37.7	
Any secondary or higher	12.0	11.0	
Father’s occupation			.42
Farmer	90.6	87.5	
Jobless	0.0	1.6	
Other	7.4	11.0	
Father’s education			.68
Informal education, never attended school	13.6	14.1	
Lower primary (1–4)	33.3	28.1	
Upper primary (5–8)	43.9	45.3	
Any secondary or higher	9.1	12.5	
Household assets: land, ha	0.1±0.0	0.1±0.0	.41
Household domestic asset index^a^	9.1±0.6	10.1±1.0	.36
CASHPOR housing index^b^	5.0±0.2	5.2±0.2	.03
Household food insecurity access category			.98
Food secure	13.7	17.0	
Mild food insecurity	0.9	0.4	
Moderate food insecurity	22.2	20.1	
Severe food insecurity	63.2	62.5	

Abbreviation: SE, standard error.

^a^ Household domestic asset index was calculated for all movable assets including livestock, so that each asset was assigned a weight then adjusted for age. Higher asset scores indicate higher socioeconomic status.

^b^ The CASHPOR housing index captures quality of housing by using roof, wall, and floor materials as a proxy for measuring poverty. CASHPOR scores below 5 indicate very poor housing and scores from 5 to 9 indicate poor housing.

Most children in both study groups were still breastfed at baseline (intervention 86.3%, control 83.8%), whereas few children continued to be breastfed at endline (intervention 15.3%, comparison 13.7%). Current breastfeeding status did not differ significantly by study group at baseline or endline. Mean meal frequency was low at baseline (intervention 2.6±0.4, control 2.6±0.4), remained similar at endline (intervention 2.6±0.4 meals, control 2.4±0.4 meals), and did not differ significantly by study group at either time point.

### Exposure to CHW Activities and *Gabura Amata Mubyeyi*

CHWs in both study areas continued with their usual home visits and community nutrition activities throughout the intervention period, while CHWs in the intervention areas also implemented the additional *Gabura Amata Mubyeyi* intervention components. More than 70% of mothers in both study groups reported that they had been visited at home by a CHW in the past 6 months and more than 75% had contact with a CHW in the community to discuss nutrition in the past 6 months (Supplement Table 2). Difference-in-difference estimates were 9.0 percentage points higher for CHW home visits (*P*=.02) and 10.3 percentage points higher for contact with a CHW in the community (*P*=.03) in the intervention group compared with the control group.

Table 2 shows exposure to the *Gabura Amata Mubyeyi* SBCC intervention among mothers in the intervention group. Ninety percent of mothers in the intervention group were visited at home by a CHW to discuss *Gabura Amata Mubyeyi* and they had an average of 5.3±5.1 visits. Eighty-three percent of mothers in the intervention group participated in community activities in which the CHW discussed ASFs or milk, and CHWs discussed these topics during community activities an average of 5.9±4.6 times during that period.

**TABLE 2.** Intervention Participants’ Exposure to *Gabura Amata Mubyeyi* Activities Conducted by Community Health Workers

	Home Visits (N=223), % or Mean±SD	Community Activities (N=223), % or Mean±SD
Mother participated in *Gabura Amata Mubyeyi*	90.7 (n=195^a^)	82.8 (n=178^b^)
CHW used *Gabura Amata Mubyeyi* educational materials	82.0	90.4
Type of educational materials used		
Counseling cards	64.2	66.9
Brochure	88.7	0.0
Poster	18.2	0.0
Topics CHW discussed		
Importance of animal source foods for children and mothers	90.3	98.3
Children should drink 1 cup of cow’s milk per day	74.4	77.8
Introduce cow’s milk at 12 months	73.4	79.4
No. of home visits or community activities during which CHW talked about these topics	5.3±5.1	5.9±4.6

Abbreviation: CHW, community health worker.

^a^ Of the mothers who participated in *Gabura Amata Mubyeyi* home visits.

^b^ Of mothers who participated in *Gabura Amata Mubyeyi* community activities.

### Impact on Mothers’ Knowledge and Awareness

At endline, more mothers in the intervention group compared with the control group were able to name the ASFs, for instance, milk (90.1% vs. 81.7%, *P*=.03), fish (61.0% vs. 50.7%, *P*=.04), and eggs (82.1% vs. 70.8%, *P*=.01), and more knew that children should not start to receive cow’s milk until 12 months of age (41.7% vs. 18.7%, *P*<.001) (Table 3). Mothers in the intervention group also had greater awareness than mothers in the control group that they should feed their child ASFs (76.2% vs. 62.1%, *P*=.01), feed the child 1 cup of cow’s milk per day (20.6% vs. 7.8%, *P*<.001), and introduce cow’s milk at 12 months of age (35.9% vs. 11.0%, *P*<.001). We found no differences between the study groups in mothers’ knowledge of the number ASFs a child should consume daily, main nutrients in cow’s milk, and quantity of cow’s milk that a child should drink daily.

**TABLE 3.** Differences in Mothers’ Knowledge and Awareness Related to Milk and Other Animal Source Foods at Endline

	Intervention (N=223), %	Control (N=219), %	Difference, %	*P* Value
Types of food considered to be ASFs				
Milk	90.1	81.7	8.4	.03
Meat (beef, goat, chicken, pork)	91.0	84.5	6.6	.07
Fish	61.0	50.7	10.3	.04
Eggs	82.1	70.8	11.3	.01
No. of types of ASFs a child should eat daily				
0	5.4	3.7	1.7	.44
1	10.3	14.2	−3.8	.26
2 or more	74.9	73.1	1.8	.74
Main nutrients in cow’s milk				
Calcium	4.0	5.5	−1.4	.48
Protein	31.4	27.4	4.0	.36
Fat	5.4	7.3	−1.9	.45
Carbohydrates	18.4	13.2	5.1	.18
Quantity of cow’s milk a child should drink each day				
1 cup or more	87.0	88.1	−1.1	.77
Age when a child is old enough to receive cow’s milk				
12 months or older	41.7	18.7	23.0	.00
Awareness				
Feed the child ASFs	76.2	62.1	14.1	.01
Feed the child 1 cup or 240 mL of cow’s milk every day	20.6	7.8	12.9	.00
Introduce cow’s milk at age 12 months	35.9	11.0	24.9	.00

Abbreviation: ASF, animal source food.

At endline, more mothers in the intervention group could name the ASFs, and more knew that children under 12 months should not receive cow’s milk.

### Household Milk Use

Nearly half of the households in both groups reported that they never used the milk produced by their cow either because the production is too low and they leave the milk for the calf or the cow has not calved (intervention, 42.3% baseline, 48.4% endline; control, 42.5% baseline, 49.8% endline). Among households that used the milk from their cow, 58%–75% kept all their morning milk and 79%–87% kept all their evening milk, indicating that an important portion of the households sold some or all of their milk, especially milk collected in the morning (Supplement Table 3). The percentage of households that kept or sold their milk did not differ by study group. Among households that reported keeping some or all of their milk, mean milk production in both groups was approximately 1 L of milk at baseline and 1.5 L at endline (data not shown). We found no difference in milk production by group at either time point.

### Impact on Children’s ASF Consumption, Milk Consumption, and Dietary Diversity

Approximately half of children in both study groups had not consumed fresh cow’s milk during the past week at endline. Among children who consumed fresh milk, the difference-in-difference estimate for consumption of fresh cow’s milk 2 or more times per week was 8.0 percentage points higher in the intervention group compared with the control group, although the difference was not statistically significant (adjusted *P*=.17) (Table 4). Children in the intervention group had increased odds of consuming cow’s milk 2 or more times per week if their mothers recalled hearing that children should drink 1 cup of cow’s milk per day during a CHW’s home visit (odds ratio [OR] 2.1, 95% confidence interval [CI] 1.1, 3.9) or a community activity (OR 2.0, 95% CI 1.2, 3.5).

**TABLE 4.** Impact of *Gabura Amata Mubyeyi* on Children’s Animal Source Food (ASF) Consumption, Milk Consumption, and Dietary Diversity

	Baseline (T1)		Endline (T2)						
	Intervention (N=234), %	Control (N=228), %	Intervention (N=223), %	Control (N=219), %	Intervention (T2 − T1)^a^	Control (T2 − T1)^a^	DiD Impact Estimate^a^	*P* Value	Adj. *P* Value
ASF consumption (24-hour recall)^b^	55.6	47.8	40.8	36.5	−14.7	−11.3	−3.5	.63	.68
Dairy consumption (24-hour recall)^c^	44.0	36.4	31.4	26.0	−12.6	−10.4	−2.3	.86	.94
Fresh cow’s milk consumption (24-hour recall)	9.4	7.5	30.5	25.1	21.1	17.7	3.4	.95	.84
Fresh cow’s milk consumption (7-day recall)									
Never	54.7	56.6	48.0	51.6	−6.7	−5.0	−1.7	.77	.73
1 time per week	2.6	0.4	2.2	6.4	−0.3	6.0	−6.3	.02	.02
2 or more times per week	42.7	43.0	49.8	42.0	7.0	−1.0	8.0	.20	.17
Minimum dietary diversity	51.3	44.3	47.1	40.2	−4.2	−4.1	−0.1	.99	.99

Abbreviations: ASF, animal source food; DiD, difference-in-difference.

^a^ Percentage point difference.

^b^ ASF consumption includes meat, poultry, fish, eggs, and dairy, including fresh and powdered milk.

^c^ Dairy consumption includes fresh and powdered milk, yogurt, and cheese, but very little yogurt or cheese was consumed by children in this study (see Supplemental Figure 1).

Children were more likely to consume cow’s milk at least twice per week if their mothers recalled hearing that children should drink 1 cup of cow’s milk per day.

The intervention was not associated with children’s ASF consumption (24-hour recall), dairy consumption (24-hour recall), fresh cow’s milk consumption (24-hour recall), or minimum dietary diversity. ASF consumption and dairy consumption decreased in both groups from baseline to endline, whereas fresh cow’s milk consumption (24-hour recall) increased by 21.1% in the intervention group and 17.7% in the control group. The specific types of ASFs consumed by the children in both study groups at baseline and endline are shown in Supplement Figure 1. Dietary diversity was 3.4±0.1 food groups in the intervention group and 3.3±0.1 in the control group at baseline; it did not change significantly from baseline to endline.

## DISCUSSION

In this study, we designed an SBCC intervention that was implemented by CHWs who promoted the consumption of ASFs, especially cow’s milk, among children in households that received a cow through the Girinka program in 2 districts of Rwanda. We detected impacts of the intervention on mothers’ ASF knowledge and awareness and an increased odds of more frequent milk consumption among children whose mothers were exposed to the intervention, but no effects on the prevalence of milk consumption during the past 24 hours or minimum dietary diversity. We hypothesized that the intervention would work through the own-production pathway and that increased maternal knowledge would lead to increased consumption of household-produced milk and subsequently to increased dietary diversity. The most likely explanations for the lack of impacts of the intervention on nutrition outcomes were the low milk production of the cows and the high level of food insecurity and poverty among the participants, which led to competing needs for household resources. Cows in nearly half of the households were not productive enough for the household to use the milk, and up to 40% of households with enough milk sold some or all of it. This finding suggests that milk is an important source of income for these families and SBCC alone may not modify milk use patterns in Girinka households at current levels of milk production. This aligns with results from other studies showing that SBCC is not sufficient to change nutrition outcomes in households with poor food security.^41^^,^^42^ It is also congruent with agriculture-nutrition pathways indicating that income and sufficient resources for food expenditures are needed for agricultural programs to have nutrition impacts.^22^^,^^25^

The lack of impacts of the intervention on nutrition outcomes was likely due to low milk production and high levels of food insecurity and poverty.

This study demonstrated that it is feasible for government health staff to train and supervise CHWs to implement an ASF SBCC intervention. Mothers in the intervention group reported frequent contacts with CHWs as part of this intervention both through home visits and community activities related to nutrition. CHWs used the SBCC materials and transmitted the key messages. These activities resulted in increases in some aspects of maternal knowledge and awareness related to milk. Mothers in both study groups had high levels of knowledge about some topics, including which foods constitute ASFs, the number of ASFs that should be eaten daily, and the quantity of milk that should be given to a child daily. Maternal knowledge about giving children 1 cup of milk per day most likely came from the Rwanda Agriculture Board’s One Cup of Milk Per Child program, which has provided milk to school children since 2011.^34^ Mothers’ knowledge and awareness about introducing cow’s milk at 12 months was lower before the intervention than for other topics and it increased during this study.

We found several notable changes in children’s dairy consumption in both study groups during this study. Children’s fresh cow’s milk consumption in the past 24 hours increased greatly from baseline to endline, while dairy consumption decreased. The increase in fresh cow’s milk consumption in both study groups may be partly related to differences in the timing of data collection at baseline and endline. Baseline data were collected across long rains, long dry season, and short rains, whereas endline data were collected during the latter part of short rains, when fodder is more plentiful. However, the differences in mean milk production from baseline to endline were small. The change in type of milk consumed by children from baseline to endline was more likely related to their use of a locally produced fortified maize-soy blend containing milk powder (known as *Shisha Kibondo*), which is provided for free at health facilities to low-income families with children <24 months.^43^^,^^44^ Most of the children in our study were <24 months at baseline, so *Shisha Kibondo* accounted for the majority of their milk, dairy, and ASF consumption at baseline. As the children grew and no longer received *Shisha Kibondo*, their consumption of fresh cow’s milk increased and accounted for most of their dairy and ASF consumption. The shift away from *Shisha Kibondo* consumption also explains why ASF and dairy consumption decreased over time. Children in both study groups had a higher prevalence of dairy consumption compared with children aged 6–23 months in the Rwanda Demographic and Health Survey^31^ and to children among livestock-owning households in Tanzania.^11^ However, given that all households in this study had a cow, children’s milk consumption was still low, with no milk consumption being reported for about half of the children during the past week. Interestingly, most children in this study either received fresh cow’s milk 2 or more times per week or not at all, which may indicate that when households have fresh milk available, they do give it to children.

### Strengths and Limitations

The strengths of this study were a cluster-randomized design and a well-designed SBCC intervention based on formative research. This study also had several limitations. The intervention was originally planned for 12 months but had to be shortened because of challenges in getting approvals from various government agencies. Our baseline data were collected in batches over several months because we received lists of potentially eligible households at different times and needed to collect data before the children were no longer eligible. As a result, our baseline and endline data were not collected during the same time of year. However, the difference in timing of data collection did not appear to be related to milk production because we found no differences in milk production between study groups. To stay within budget constraints, our study was powered for a 15-percentage point difference. This explains why the 8-percentage point higher frequency of weekly milk consumption detected in this study was not statistically significant and indicates that future studies should use a smaller percentage point difference to estimate the sample size needed to detect between group differences in milk consumption and dietary diversity.

BOX 1 **Key Messages in the *Gabura Amata Mubyeyi* Social and Behavior Change Communication Materials**

Importance and benefits of animal source foods (ASFs) and milk consumption for children aged 1–3.5 years:

- Milk is rich in calcium needed for bone formation and has fat and protein needed for children to grow well.
- ASFs provide multiple micronutrients simultaneously. For example, food such as liver contains iron and vitamin A.

Appropriate quantities of ASFs and cow’s milk to be consumed by children aged 1–3.5 years:

- Children aged 6–11 months should be fed at least 1 portion of ASFs, such as eggs, meat, fish, or chicken, to meet their daily nutrient needs, in addition to continued breastfeeding.
- Children 1 year and older should drink at least 1 cup (240 mL) per day or eat at least 1 portion of other ASFs.
- Mothers who are not breastfeeding their children should seek advice from community health workers or health providers on introducing ASFs and cow’s milk to their young children.

Appropriate time to introduce cow’s milk and ASFs to young children:

- Children aged 6 months should be given breast milk and introduced to ASFs such as meat, poultry, fish, and eggs, but not cow’s milk.
- Children aged 12 months should be introduced to cow’s milk into their daily diet.
- A child should continue to be breastfed even after cow’s milk is introduced. Breast milk protects a child from illnesses and reduces the risk of malnutrition.
- Household cow’s milk production should be used primarily to feed children and mothers at least 1 cup of milk each per day to improve maternal and child nutrition.

## CONCLUSION

In conclusion, this study found effects of an ASF SBCC intervention on maternal knowledge and awareness related to milk consumption, and intervention exposure was associated with increased odds of children’s milk consumption 2 or more times per week. Although we hypothesized that the SBCC intervention would increase milk consumption through the own-production pathway, more than half of the households in this study either had inadequate production for human consumption or sold their milk. This finding indicates that interventions to increase household milk production, influence decision making around retention of milk for home consumption, and influence how the proceeds of milk sales are used for household nutrition could be impactful, as was shown in an agriculture-nutrition program in Burkina Faso.^45^ Insufficient milk production by Girinka cows and the need for some households to sell their milk also suggests that the Girinka program may need to add other components or supporting activities that would assist households to increase milk production and/or to keep more of the milk that they produce. It also indicates that SBCC may need to be tailored to support increased ASF consumption through the agriculture-nutrition “income” pathway. The messages would need to focus on cost-effective ways to improve children’s diets with the income from cow’s milk sales and potentially on increasing women’s control over resources and decision making related to food purchases. Finally, the low levels of knowledge on some ASF topics at endline, despite large differences between groups (e.g., introducing milk at 12 months), indicate that a longer duration SBCC intervention may be needed to increase knowledge and modify social norms. This process is already underway as the National Child Development Agency has incorporated the *Gabura Amata Mubyeyi* messages into the recently revised national CHW counseling cards and is training CHWs on their use.

## Supplementary Material

21-00082-Flax-Supplement.pdf

## Translation

**En français** **Intervention de communication sur le changement social et comportemental par rapport à l’alimant d’origine animale parmi les bénéficiaires du transfert de bovins Girinka au Rwanda: une évaluation randomisée en**

**Principaux résultats**

- L’exposition à l’intervention était associée à une probabilité accrue que les enfants consomment du lait de vache 2 fois ou plus par semaine.
- Environ la moitié de la fréquence de consommation de lait de vache chez les enfants était limitée par une production ou une vente inadéquate du lait produit par les ménages.
- La CCCS n’a pas eu d’influence sur le pourcentage de ménages qui ont gardé ou vendu leur lait, démontrant ainsi que la CCCS seule ne suffit pas à modifier les résultats nutritionnels dans les ménages ayant une faible sécurité alimentaire.

**Implications Clés:**

- Les agents de la santé communautaire (ASC) ont mis en œuvre avec succès l’intervention et les messages de la CCCS ont été intégrés dans les cartes nationales de conseil des ASCs récemment révisées
- CCCS pour cette population cible devrait être mise en œuvre pour une période plus longue et adaptée pour discuter de la gestion financière ou des choix alimentaires avec un budget limité. Une formation d’accompagnement ou d’autres activités visant à aider les ménages qui reçoivent des vaches sont nécessaires pour assurer une production adéquate de lait pour la consommation domestique.
- s niveaux élevés d’insécurité alimentaire grave dans cette population peuvent avoir limité le potentiel de la CCCS d’améliorer la diversité alimentaire et d’améliorer plus considérablement la fréquence de la consommation de lait de vache.

**RÉSUMÉN**

Les aliments d’origine animale, y compris le lait de vache, contiennent des nutriments essentiels et contribuent à une alimentation saine, mais la fréquence de consommation est faible chez les enfants dans les pays à revenu faible ou intermédiaire. Nous avons émis l’hypothèse qu’une intervention de communication sur le changement de comportement et social (CCCS) mise en œuvre par les agents de santé communautaires (ASC) lors des visites mensuelles à domicile et des activités au niveau communautaire augmenteraient la consommation de lait par les enfants et la diversité alimentaire dans les ménages qui ont reçu une vache du programme de transfert de bétail Girinka du gouvernement du Rwanda. Nous avons testé l’intervention CCCS sur 9 mois dans une cohorte d’enfants âgés de 12 à 29 mois au départ dans des cellules administratives aléatoirement affectées aux groupes d’intervention ou témoin. La plupart des mères du groupe d’intervention ont été exposées aux visites à domicile par les ASCs (90,7 %) et aux activités communautaires (82,8 %). À la fin de l’intervention, plus de mères dans le groupe d’intervention comparé au groupe témoin savaient que le lait de vache était un aliment d’origine animale (90,1 % contre 81,7 %, P = 0,03) et qu’il pouvait être introduit aux enfants à 12 mois (41,7 % contre 18,7 %, P<0,001). Plus de mères dans le groupe d’intervention comparé au groupe témoin savaient qu’elles devraient nourrir leur enfant d’aliment d’origine animale (76,2 % contre 62,1 %, P = 0,01) et leur donner une tasse de lait de vache par jour (20,6 % contre 7,8 %, P<0,001). La consommation de lait de vache frais par les enfants 2 fois ou plus de fois par semaine a augmenté dans le groupe d’intervention, mais pas de manière significative (8,0 points de pourcentage, P = 0,17); la diversité diététique minimale n’a pas changé. Les enfants du groupe d’intervention avaient une probabilité accrue de consommer du lait de vache 2 fois ou plus par semaine si leur mère se souvenait d’avoir entendu dire que les enfants devraient boire une tasse de lait de vache par jour lors d’une visite à domicile d’une ASC [odds ratio (OR) 2,1, intervalle de confiance (IC) 95 % (1.1, 3.9)] ou d’une activité communautaire [OR 2,0, IC à 95 % (1.2, 3.5)]. Environ la moitié des enfants n’ont pas eu de lait au cours de la dernière semaine parce que leur ménage produisait peu de lait ou vendait ce qui était produit l. Dans les ménages pauvres qui reçoivent un transfert de bétail, des stratégies visant à adapter davantage la CCCS et à augmenter la production de lait de vache peuvent être nécessaires pour obtenir une augmentation plus importante de la fréquence de la consommation de lait chez les enfants.

**Peer Reviewed**

**First published online:** August 4, 2021.
