# Supplementary material for: Animal Source Food Social and Behavior Change Communication Intervention Among Girinka Livestock Transfer Beneficiaries in Rwanda: A Cluster Randomized Evaluation
Source: Glob Health Sci Pract. 2021 Sep 30;9(3):640–53. doi: 10.9745/GHSP-D-21-00082 (PMC8514034; doi:10.9745/GHSP-D-21-00082)
Supplement: 21-00082-Flax-Supplement.pdf [file 21-00082-Flax-Supplement.pdf]

Supplement to: Flax VL, Ouma E, Izerimana L, et al. Animal source food social and behavior change communication intervention among Girinka livestock transfer beneficiaries in Rwanda: a cluster randomized evaluation. *Glob Health Sci Pract*. 2021;9(3). https://doi.org/10.9745/GHSP-D-21-00082

Supplemental Figure 1. Types of Animal Source Foods (ASFs) Consumed by Children (24-Hour Recall)

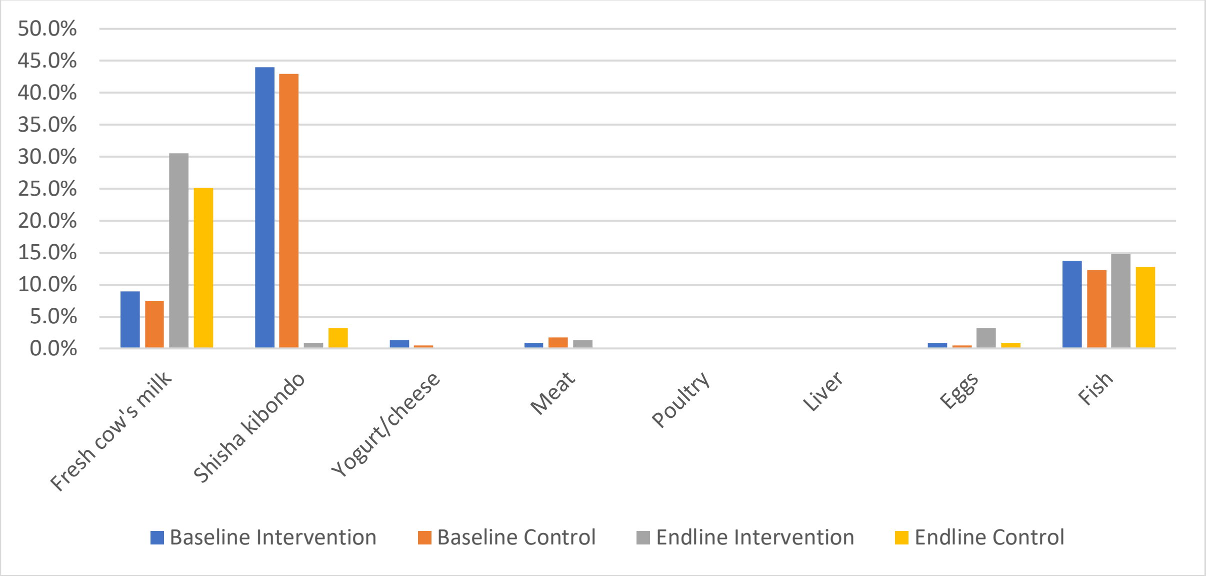

Shisha kibondo is a maize-soy blend with dry milk powder provided for free to poor households with a child aged 6–23 months.

Fish are usually small dried fish.

Supplement to: Flax VL, Ouma E, Izerimana L, et al. Animal source food social and behavior change communication intervention among Girinka livestock transfer beneficiaries in Rwanda: a cluster randomized evaluation. *Glob Health Sci Pract*. 2021;9(3). https://doi.org/10.9745/GHSP-D-21-00082

SUPPLEMENTAL TABLE 1. Summary of Sample Size Calculations

	Hypothesized Levels				Power 80%; Significance Level 5%			Across Districts (per Group)	
	No SBCC Intervention		SBCC Intervention						
Response	Baseline	Endline	Baseline	Endline	Unadjusted, N (per group)	Average cluster size (HH/cell)	Intracluster Correlation	Adj. N	No. of cells
Dietary diversity, %	24	29	24	44	158	4	0.1	206	52
Milk consumption, %	20	20	20	35	134	4	0.1	175	44

Abbreviations: HH, household; SBCC, social and behavior change communication.

We calculated sample sizes for 2 child outcomes—minimum dietary diversity (consumption of ≥4 food groups in the last 24 hours) and milk consumption in the last 24 hours—based on comparison of the changes in these parameters between baseline and endline. A 2-sided 2-sample binomial calculation was used to estimate sample size for dietary diversity and milk consumption. We used the Rwanda 2014–2015 Demographic and Health Survey data to calculate the prevalence of minimum dietary diversity (24%) and milk consumption (20%) in our target age group.[1] Minimum dietary diversity required the largest sample size, so was used as the sample size for the study. To detect a 15-percentage point difference between groups[2] (i.e., at endline no SBCC intervention 29% and SBCC intervention 44%) with 80% power and alpha=0.05, required 208 households per arm assuming an average cluster size of 4 households per cell, an intracluster correlation of 0.10, and a design effect of 1.3. We added 10% to the sample to account for attrition, resulting in 229 households per arm and a total baseline sample size of 458.

[1] National Institute of Statistics of Rwanda (NISR) [Rwanda], Ministry of Health (MOH) [Rwanda], ICF International. *Rwanda Demographic and Health Survey 2014–15: Final Report*. NISR, MOH, and ICF International; 2016. Accessed July 9, 2021. https://dhsprogram.com/pubs/pdf/FR316/FR316.pdf

[2] Kuchenbecker J, Reinbott A, Mtimuni B, Krawinkel MB, Jordan I. Nutrition education improves dietary diversity of children 6-23 months at community-level: results from a cluster randomized controlled trial in Malawi. *PLoS One*. 2017;12:e0175216.

Supplement to: Flax VL, Ouma E, Izerimana L, et al. Animal source food social and behavior change communication intervention among Girinka livestock transfer beneficiaries in Rwanda: a cluster randomized evaluation. *Glob Health Sci Pract*. 2021;9(3). https://doi.org/10.9745/GHSP-D-21-00082

SUPPLEMENTAL TABLE 2. Participants' Exposure to Community Health Workers' (CHWs') Usual Home Visits and Community Nutrition Activities in the Past 6 Months[a]

	Baseline (T1)		Endline (T2)					
	Intervention (N=234), %	Control (N=228), %	Intervention (N=223), %	Control (N=219), %	Intervention (T2 − T1)[b]	Control (T2 − T1)[b]	DiD Impact Estimate[b]	*P*-Value
Visited at home by a CHW	76.9	75.0	86.1	72.5	6.5	−2.5	9.0	.02
Any contact with CHW in the community to discuss nutrition	80.8	79.8	89.6	78.4	8.9	−1.4	10.3	.03

[a]This table shows participants' exposure to CHWs conducting home visits and community nutrition activities. For both study groups at baseline, this represents usual CHW activities. At endline, it continues to represent usual CHW activities for the control group but represents both usual CHW activities and *Gabura Amata Mubyeyi* activities for the intervention group. Details of *Gabura Amata Mubyeyi* exposure in the intervention group are shown in Table 3.

[b]Percentage point difference.

Supplement to: Flax VL, Ouma E, Izerimana L, et al. Animal source food social and behavior change communication intervention among Girinka livestock transfer beneficiaries in Rwanda: a cluster randomized evaluation. *Glob Health Sci Pract*. 2021;9(3). https://doi.org/10.9745/GHSP-D-21-00082

SUPPLEMENTAL TABLE 3. Usual Use of Household Produced Milk From Girinka Cows Among Households That Reported Ever Using the Milk

	Baseline (T1)		Endline (T2)					
	Intervention (N=135), %	Control (N=131), %	Intervention (N=115), %	Control (N=110), %	Intervention (T2 − T1)[a]	Control (T2 − T1)[a]	DiD Impact Estimate[a]	*P*-Value
Morning milk								
Keep milk entirely for household consumption	72.2	58.3	64.8	63.5	−7.4	5.1	−12.5	.11
Use milk partly for consumption, partly for sale	19.0	29.2	26.9	28.8	7.8	−0.3	8.1	.20
Use milk entirely for sale	8.7	12.5	8.3	7.7	−0.4	−4.8	4.4	.48
Evening milk								
Keep milk entirely for household consumption	79.3	82.4	81.3	86.7	2.0	4.3	−2.3	.85
Use milk partly for consumption, partly for sale	18.3	15.3	17.2	11.7	−1.1	−3.6	2.5	.77
Use milk entirely for sale	2.4	2.4	1.6	1.7	−0.9	−0.7	−0.2	.90

Abbreviation: DiD, difference-in-difference.

[a]Percentage point difference.
